# Pearson's Dentists' Appointment Book

**Published:** 1889-02

**Authors:** 


PEARSON'S DENTISTS' APPOINTMENT BOOK is one of the neatest and most convenient publications for such a purpose that has ever been issued. It is not only attractive in appearance, but can be carried in the vest pocket. Its management of dates, &c., is all that could be desired.

